# Comorbidity and health services' usage in children with autism spectrum disorder: a nested case–control study

**DOI:** 10.1017/S2045796020000050

**Published:** 2020-01-28

**Authors:** Yotam Dizitzer, Gal Meiri, Hagit Flusser, Analya Michaelovski, Ilan Dinstein, Idan Menashe

**Affiliations:** 1 Joyce & Irving Goldman Medical School, Faculty of Health Sciences, Ben-Gurion University of the Negev, and Clinical Research Center, Soroka University Medical Center, Beer-Sheva, Israel; 2 Pre-School Psychiatry Unit, Soroka University Medical Center, Beer-Sheva, Israel; 3 Child Development Center, Soroka University Medical Center, Beer-Sheva, Israel; 4 Psychology Department, and Cognitive and Brain Sciences Department, Ben-Gurion University of the Negev, Beer-Sheva, Israel; 5 Zlotowski Center for Neuroscience, Ben-Gurion University of the Negev, Beer-Sheva, Israel; 6 Public Health Department, Faculty of Health Sciences, Ben-Gurion University of the Negev, Beer-Sheva, Israel

**Keywords:** Autism spectrum disorder, comorbidity, health care, health services

## Abstract

**Aims:**

Children with autism spectrum disorder (ASD) tend to suffer from various medical comorbidities. We studied the comorbidity burden and health services' utilisation of children with ASD to highlight potential aetiologies and to better understand the medical needs of these children.

**Methods:**

In this nested case–control study, ASD cases and controls – matched by age, sex and ethnicity in a 1:5 ratio – were sampled from all children born between 2009 and 2016 at a tertiary medical centre. Data were obtained from the hospital's electronic database. Comorbid diagnoses were classified according to pathophysiological aetiology and anatomical/systemic classification of disease. Standard univariate and multivariate statistics were used to demonstrate comorbidities and health services' utilisation patterns that are significantly associated with ASD.

**Results:**

ASD children had higher rates of comorbidities according to both pathophysiological and anatomical/systemic classifications (*p* < 0.001). The most marked significant differences were observed for: hearing impairments (OR = 4.728; 95% CI 2.207–10.127) and other auricular conditions (OR = 5.040; 95% CI 1.759–14.438); neurological (OR = 8.198; 95% CI 5.690–11.813) and ophthalmological (OR = 3.381; 95% CI 1.617–7.068) conditions; and ADD/ADHD (OR = 3.246; 95% CI 1.811–5.818). A subgroup analysis revealed a more profound case–control difference in anaemia rates among girls than in boys (OR = 3.25; 95% CI 1.04–10.19 *v*. OR = 0.74; 95% CI 0.33–1.64 respectively) and an opposite trend (larger differences in males than in females in cardiovascular diseases (OR = 1.99; 95% CI 1.23–3.23 *v*. OR = 0.76; 95% CI 0.17–3.45, respectively)). In addition, larger case–control differences were seen among Bedouin children than in Jewish children in a number of medical comorbidities (Breslow–Day test for homogeneity of odds ratio *p*-value <0.05). Finally, we found that children with ASD tended to be referred to the emergency department and to be admitted to the hospital more frequently than children without ASD, even after adjusting for their comorbidity burden (aOR = 1.28; 95% CI 1.08–1.50 and aOR = 1.28; 95% CI 1.11–1.47 for >1 referrals and admissions per year, respectively).

**Conclusions:**

The findings of this study contribute to the overall understanding of comorbid conditions and health services' utilisation for children with ASD. The higher prevalences of comorbidities and healthcare services' utilisation for children with ASD highlight the additional medical burden associated with this condition.

## Introduction

Autism spectrum disorder (ASD) comprises a group of neurodevelopmental disorders that share common fundamental impairments in social interaction, verbal and non-verbal communication, and repetitive behaviours (Masi *et al*., 2017). The aetiology of these disorders is unknown and probably involves both genetic and non-genetic (environmental) factors (Newschaffer *et al*., 2007; Kim and Leventhal, 2015; Lyall *et al*., 2017). In recent years, the clinical manifestations of ASD – beyond those included in the core Diagnostic and Statistical Manual of Mental Disorders (DSM) criteria – have been gaining increasing attention (Xue *et al*., 2008; Bauman, 2010; Coury, 2010; Kohane *et al*., 2012). For example, studies examining co-occurring morbidities (comorbidities) in children with ASD have revealed a tendency for children with ASD to suffer from other disorders, including neurological disorders (such as epilepsy (Simonoff *et al*., 2008; Weissman and Bates, 2010; Jeste, 2011)), sleep disorders (Mouridsen *et al*., 1999; Jeste, 2011), motor impairments (Jeste, 2011; Maski *et al*., 2011) and psychiatric comorbidities (such as schizophrenia (Zheng *et al*., 2018), social anxiety disorder, attention-deficit/hyperactivity disorder (ADHD) and oppositional defiant disorder (Simonoff *et al*., 2008; Antshel *et al*., 2013)). Among these studies, some have demonstrated a relationship between the severity of the neurocognitive impairment and psychiatric comorbidities in children with ASD (Weissman and Bates, 2010). In addition, alongside these studies, attention is gradually being focused on non-psychiatric or non-neurocognitive clinical comorbidities in children with ASD (Horvath and Perman, 2002; Richdale and Schreck, 2009; Charlot *et al*., 2011; Doshi-Velez *et al*., 2014), including gastrointestinal (GI) and auditory disorders (Horvath and Perman, 2002).

The very extensive body of work on children with ASD indicates that the above comorbidities with ASD constitute a high burden on health services. One of the earliest studies exploring this idea was that of Kohane *et al*. (2012), who examined the comorbidity burden of children and young adults with ASD admitted to four different hospitals in the Boston area. They found that in comparison with the general admitted population, there was a higher burden in the ASD population of specific comorbidities, including inflammatory bowel disease and other GI disorders, and diabetes mellitus type 1. That study was complemented by another from the same group showing a correlation between psychiatric and GI disorders in children with ASD (Kohane *et al*., 2012; Doshi-Velez *et al*., 2014). A recent study assessing clustered groups of children with ASD and co-occurring medical comorbidities showed high rates of auditory, immune and GI comorbidities, with similar longitudinal patterns of prevalence between GI and immune and between seizure and sleep disorders as well (Vargason *et al*., 2019). It thus appears that understanding this comorbidity burden is essential for a number of reasons – for the light it can throw on the underlying aetiologies associated with ASD; for stratifying the risk of various conditions across individuals with ASD; and for aiding healthcare suppliers in planning for meeting the needs of the ASD population.

A cardinal – and largely understudied – issue that is clearly related to comorbidities is the actual utilisation of health services by children with ASD and the attendant expenditures. Although it is known that children with ASD require more extensive educational and behavioural services than most other children (Chambers *et al*., 2003), their needs for medical services remain to be quantified. The sparse information that is available on this issue is somewhat out of date: In 2006, Liptak *et al*. (2006) reported on the medical utilisation and health-related expenditures of children with ASD. They found that children with ASD have a substantial burden of medical illness, which is manifested in health-related activities, such as more frequent outpatient visits to the doctor *v*. those for children in general. In another report that appeared in the same year, Croen *et al*. (2006) found that the utilisation and costs of health care are substantially higher for children with ASD than for children without ASD.

Illuminating chronic diagnoses that are associated with ASD will shed light on potential biological mechanisms underlying these co-morbid conditions. Furthermore, revealing differences in healthcare utilisation between children with and without ASD and understanding the reasons for these differences may help reducing the medical and social burden from children with ASD and their families. Therefore, we set out to assess the comorbidity burden and health care utilisation patterns in children with ASD in comparison with children without ASD, as reflected in the electronic health records of the Soroka University Medical Center (SUMC) in Beer-Sheva, Israel.

## Methods

### Design

This is a nested case–control study. Cases and controls were ascertained from all children born at SUMC between the years 2009 and 2016 whose families were listed to be residents of Israel's southern region, the Negev. All children were members of the Clalit Health Services, the largest HMO in Israel, which insures and provides health care services to ~75% of the population in southern Israel. Cases were children from the regional database of the Negev Autism Center (NAC) who were diagnosed with ASD according to the DSM-V criteria at the Preschool Psychiatry Unit at SUMC as described previously (Meiri *et al*., 2017) (*n* = 459). The control group comprised children without a diagnosis of ASD or of any known genetic syndrome, such as Trisomy 21 or Fragile X syndrome. Children in the control group were matched to ASD cases on the basis of age, sex and ethnicity (Jewish/Bedouin), in a 1:5 case–control ratio to prevent potential bias in comorbidity rates between these groups according to these confounders (*n* = 2285; 99.6% of the intended sample size of 459 × 5 = 2295 who met both inclusion and exclusion criteria of the study).

Notably, SUMC is the only tertiary and the largest medical centre in southern Israel. It is also the main medical centre which provides health services to members of the Clalit HMO. Thus, the majority of children from southern Israel who are members of the Clalit HMO were born and continue to receive medical care at SUMC as needed.

### Medical data collection

Data for the study covered demographic characteristics and past medical history, including diagnoses given by primary-care physicians in the community setting, number of visits to the community clinic, details of referrals to the Emergency Department of SUMC, number of admissions to the paediatric wards at SUMC (including length of hospitalisation during those admissions), and visits to the doctor in the hospital outpatient clinics. All data were obtained from two primary databases of computerised medical records at SUMC – designated *Chameleon* and *Ofek*. The *Chameleon* database contains all the medical records for patients referred to the Emergency Department and admitted to the hospital from 2012, including clinical data at admission and discharge, laboratory and imaging results, diagnostic and therapeutic procedures and surgeries performed during hospitalisation, and all medications prescribed during hospitalisation. The *Ofek* database houses all medical data for every patient insured by the *Clalit* HMO documenting, among other things, all diagnoses received during visits to the primary-care physician in the community setting, visits to a specialist physician, referrals to the emergency department and during admission to the hospital. The *Ofek* database also contains the medical records regarding referrals to the Emergency Department and admissions to the hospital prior to 2012, before *Chameleon* had been set up at SUMC. Medical information was collected independently for all children, beginning at their date of birth and ending in July 2017. Due to the medical documentation structure of the *Ofek* database, it comprises all recorded medical data for children included in this study, from birth until data retrieval, including situations where families had moved to other regions of Israel. The only exceptions are children who stopped using the Israeli medical system (e.g. children of families who moved out of the country). However, these events are extremely rare, especially in the relatively stable population of southern Israel.

### Comorbidity classification

To classify and quantify the different comorbidities, we first summarised all the diagnoses made by the primary-care physicians for the ASD and control groups. Next, we clustered these diagnoses into groups of diseases based on the International Classification of Diseases (ICD)-9 codes. For this purpose, we used two well-established classification methods: (1) classification by the underlying mechanism of the disease (i.e. pathophysiological classification), and (2) classification by the system or anatomical region affected by the disease (i.e. anatomical/systemic classification), as described in Encyclopedia Britannica (https://www.britannica.com/science/human-disease/Classifications-of-diseases). Complete information about disease clusters based on these two classification approaches is presented in online Supplementary Tables S1 and S2. Clustered diseases and diagnoses with prevalence lower than 0.5% within the total population were discarded due to a statistical power limitation.

### Assessment of healthcare services' utilisation

For each child in the study, we calculated the annual average for the following aspects of healthcare services' utilisation: number of visits to the primary-care physician, number of referrals to the Emergency Department and number of admissions to the hospital. We also calculated the average length of hospitalisation for each child admitted to the hospital. We compared these variables between children with and without ASD as described below.

### Statistical analysis

We compared demographic and medical variables between children with and without ASD using the appropriate univariate analyses. Specifically, nominal variables were compared using Pearson's *χ*^2^ test, continuous variables that matched parametric criteria were compared by using Student's *t*-test, and ordinal variables and continuous variables that did not match parametric criteria were compared by using Wilcoxon or Mann–Whitney *U* tests. Statistical significance was set at a *p*-value of 0.05. We also conducted subgroup analysis and used Breslow–Day test of homogeneity to examine whether case–control differences in medical comorbidities differ between groups defined by sex or ethnicity. Multivariate logistic regression analysis was used to assess the odds ratio (OR) for ASD associated with the utilisation of medical care systems, i.e. referral to the Emergency Department or hospital admission to the Paediatric Division, after taking potential confounders into account, such as the number of comorbidities diagnosed by the primary physician.

### Ethics

This study received the approval of and was supervised by the ‘Helsinki Committee’ of SUMC (SOR 222-14).

## Results

The research population included 2744 children (459 ASD cases and 2285 controls) with a mean age of 5.5 ± 1.9 (range 1.6–8.2) years. Of the children included in the study, 2216 were males (80.8%), 528 were females (19.2%), 1946 (70.9%) were Jewish and 798 (29.1%) were Bedouin, with the proportions being equally distributed between cases and controls.

### Clinical diagnoses

**Fig. 1.**
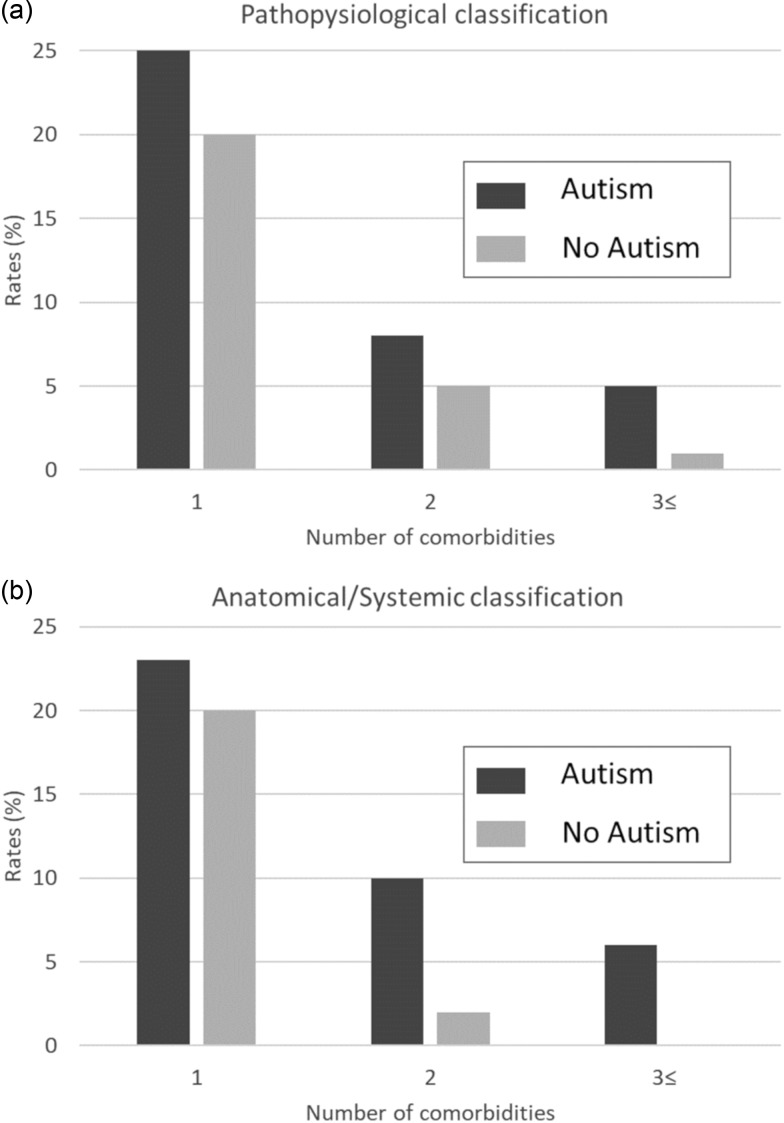
Rates of comorbidities among children with and without ASD. Rates for children with 1, 2 and 3+ comorbidities according to the pathophysiological (*a*) or anatomical/systemic classifications (*b*).

We observed a higher rate of clinical diagnoses for both the pathophysiological classification and the anatomical/systemic classification for children with ASD compared to controls (Fig. 1). Specifically, for the pathophysiological classification, 25, 8 and 5% of children with ASD had one, two and three or more comorbidities, compared to only 20, 5 and 1% of the children without ASD, respectively (Fig. 1*a*; *p* < 0.001). Similar findings were obtained for the anatomical/systemic classification, with 23, 10 and 6% of children with ASD having one, two and three or more comorbidities, compared to only 20, 2 and 0% of the children without ASD, respectively (Fig. 1*b*; *p* < 0.001).

Next, we examined the rates of comorbidities based on their pathophysiological aetiology or their anatomical/systemic localisation (Tables 1 and 2). Overall, comorbidities were more prevalent among children with ASD compared to children without ASD in both the pathophysiological (Table 1) and anatomical/systemic (Table 2) classifications. Statistically significant differences were obtained for: allergies and hypersensitivity disorders, such as asthma and atopic dermatitis (15.7 *v*. 10.7%; *p* = 0.002); congenital conditions, such as heart abnormalities (11.8 *v*. 6.2%; *p* < 0.001); hearing impairment (2.8 *v*. 0.6%; *p* < 0.001) referring to any sort of deficit in hearing, such as deafness (partial or complete), abnormal hearing test results, conductive or sensorial hearing loss and hearing complaints; infectious diseases (4.4 *v*. 2.5%; *p* = 0.023); epilepsy (2.4 *v*. 1.2%; *p* = 0.042); and overweight (8.5 *v*. 6%; *p* = 0.05). In addition, for children with ASD, significantly higher rates of comorbidities classified according to their anatomical/systemic localisation were obtained for: adenoiditis/tonsillitis (6.3 *v*. 4%; *p* = 0.026), auricular diseases such as serous otitis media (1.5 *v*. 0.3%; *p* = 0.001), cardiovascular diseases including congenital abnormalities such as atrial septal defect (ASD), ventricular septal defect and bicuspid aortic valve, pulmonary hypertension and stenosis, disturbances in cardiac electric conduction such as atrial ventricular block and abnormal cardiovascular studies such as echo cardio gram (5.7 *v*. 3.3%; *p* = 0.013), dermatological diseases including abnormal skin findings such as café au lait spots and nevus, seborrhoea and dermatitis (2 *v*. 0.8%; *p* = 0.020), endocrinological and metabolic diseases such as obesity, diabetes mellitus, hypothyroidism, gynecomastia, hirsutism and disorders of amino acid metabolism such as phenylketonuria (10 *v*. 7%; *p* = 0.025), GI diseases including reflux disease, oesophageal achalasia, duodenal atresia, inflammatory bowel diseases such as Crohn's, faecal impaction and constipation, not including infectious diseases of the GI tract such as gastroenteritis (5.4 *v*. 3%; *p* = 0.009), neurological diseases including convulsive diseases such as infantile spasms, benign rolandic epilepsy of childhood and Lennox–Gastaut syndrome, developmental delay, headaches, abnormal head circumference – micro/macrocephaly and cerebral palsy (16.6 *v*. 2.4%; *p* < 0.001), neuro-ophthalmological diseases (1.5 *v*. 0.4%; *p* = 0.002), ophthalmological conditions such as anisocoria, ambylopia, astigmatism, near and far sightedness, cataract (congenital), glaucoma (congenital), saccadic eye movement, nystagmus and tear duct obstruction, (2.6 *v*. 0.8%; *p* = 0.001), orthopaedic conditions including anatomical skeletal deformities such as genu varus and valgus, acetabular dysplasia, pectus excavatum or carinatum and scoliosis, not included traumatic orthopaedic diagnoses such as fractures and wounds (2.6 *v*. 1.2%; *p* = 0.023), and psychiatric conditions, including ADHD (4.1 *v*. 1.3%; *p* < 0.001).

**Table 1.** Diagnoses given by the primary physician in the community clinic, based on the pathophysiological classification

	Total *N* = 2744	ASD *N* = 459	w/o ASD *N* = 2285	OR (95% CI)	*p*-value
**Allergy or hypersensitivity**	**316** (**11.5%)**	**72 (15.7%)**	**244 (10.7%)**	**1.56 (1.17–2.07)**	**0.002**
Anaemia	67 (2.4%)	12 (2.6%)	55 (2.4%)	1.01 (0.58–2.05)	0.793
**Congenital**	**196 (7.1%)**	**54 (11.8%)**	**142 (6.2%)**	**2.01 (1.45–2.80)**	**<0.001**
**Developmental**	**82 (3%)**	**59 (12.9%)**	**23 (1%)**	**14.51 (8.86–23.76)**	**<0.001**
**Developmental; speech/language**	**59 (2.2%)**	**42 (9.2%)**	**17 (0.7%)**	**13.44 (7.58–23.83)**	**<0.001**
**Epilepsy**	**38 (1.4%)**	**11 (2.4%)**	**27 (1.2%)**	**2.05 (1.01–4.17)**	**0.042**
**Hearing impairment**	**27 (1%)**	**13 (2.8%)**	**14 (0.6%)**	**4.73 (2.21–10.13)**	**<0.001**
**Neurological – convulsion related**	**38 (1.4%)**	**11 (2.4%)**	**27 (1.2%)**	**2.05 (1.01–4.17)**	**0.042**
**Infectious disease**	**76 (2.8%)**	**20 (4.4%)**	**56 (2.5%)**	**1.81 (1.08–3.05)**	**0.023**
**Overweight**	**177 (6.5%)**	**39 (8.5%)**	**138 (6%)**	**1.45 (1.00–2.09)**	**0.050**
Sleep apnoea	23 (0.8%)	5 (1.1%)	18 (0.8%)	1.39 (0.51–3.76)	0.518
Trauma	23 (0.8%)	5 (1.1%)	18 (0.8%)	1.39 (0.51–3.76)	0.518
Visual impairment	14 (0.5%)	5 (1.1%)	9 (0.4%)	2.79 (0.93–8.35)	0.056

The differences in diagnoses between children with and without ASD were evaluated via Pearson's *χ*^2^ or Fisher exact tests. Statistically significant differences (*p* < 0.05) are highlighted in bold font.

**Table 2.** Diagnoses given by the primary physician in the community clinic, based on anatomical/systemic classification

	Total *N* = 2744	ASD *N* = 459	w/o ASD *N* = 2285	OR (95% CI)	*p*-value
Abdominal wall defect	43 (1.6%)	5 (1.1%)	38 (1.7%)	0.65 (0.26–1.66)	0.366
**Adenoid/tonsils**	**120 (4.4%)**	**29 (6.3%)**	**91 (4%)**	**1.63 (1.06–2.50)**	**0.026**
**Auricular diseases**	**14 (0.5%)**	**7 (1.5%)**	**7 (0.3%)**	**5.04 (1.76–14.44)**	**0.001**
**Cardiovascular**	**101 (3.7%)**	**26 (5.7%)**	**75 (3.3%)**	**1.77 (1.12–2.80)**	**0.013**
**Dermatological**	**27 (1%)**	**9 (2%)**	**18 (0.8%)**	**2.52 (1.12–5.64)**	**0.020**
**Endocrinological**	**206 (7.5%)**	**46 (10%)**	**160 (7%)**	**1.48 (1.05–2.09)**	**0.025**
**Gastrointestinal**	**94 (3.4%)**	**25 (5.4%)**	**69 (3%)**	**1.85 (1.16–2.96)**	**0.009**
Haematological	99 (3.6%)	19 (4.1%)	80 (3.5%)	1.19 (0.71–1.98)	0.503
**Neurological**	**130 (4.7%)**	**76 (16.6%)**	**54 (2.4%)**	**8.20 (5.69–11.81)**	**<0.001**
Neuromuscular	13 (0.5%)	4 (0.9%)	9 (0.4%)	2.22 (0.68–7.25)	0.174
**Ophthalmological**	**30 (1.1%)**	**12 (2.6%)**	**18 (0.8%)**	**3.38 (1.62–7.07)**	**0.001**
**Orthopaedic**	**40 (1.5%)**	**12 (2.6%)**	**28 (1.2%)**	**2.16 (1.09–4.29)**	**0.023**
Orthopaedic-fracture	15 (0.5%)	4 (0.9%)	11 (0.5%)	1.82 (0.58–5.73)	0.301
**Psychiatric – attention-deficit and/or hyperactivity**	**49 (1.8%)**	**19 (4.1%)**	**30 (1.3%)**	**3.25 (1.81–5.89)**	**<0.001**
Renal	15 (0.5%)	1 (0.2%)	14 (0.6%)	0.35 (0.05–2.70)	0.295
Respiratory	176 (6.4%)	38 (8.3%)	138 (6%)	1.40 (0.97–2.04)	0.074
Rheumatological	36 (1.3%)	8 (1.7%)	28 (1.2%)	1.43 (0.65–3.16)	0.374
Urological	76 (2.8%)	16 (3.5%)	60 (2.6%)	1.34 (0.76–2.35)	0.306

The differences in diagnoses between children with and without ASD were evaluated via Pearson's *χ*^2^ or Fisher exact tests. Statistically significant differences (*p* < 0.05) are highlighted in bold font.

### Subgroup analysis

We also examined case–control differences in rates of comorbidities based on their pathophysiological aetiology or their anatomical/systemic localisation in subgroups of our samples defined by sex and ethnicity (online Supplementary Tables S3–S6). Examining these rates in boys and girls separately revealed that anaemia was significantly more prevalent in female cases *v*. female controls, whereas no such case–control difference was seen in males (OR = 3.25; 95% CI 1.04–10.19 *v*. OR = 0.74; 95% CI 0.33–1.64, respectively; Breslow–Day *p*-value = 0.029). An opposing trend was seen in cardiovascular diseases that was more prevalent in male cases compared to male control but not among females (OR = 1.99; 95% CI 1.23–3.23 *v*. OR = 0.76; 95% CI 0.17–3.45, respectively; Breslow–Day *p*-value = 0.019). No other case–control differences were seen between the two sexes.

Examining these rates in Jewish and Bedouin children separately revealed case–control differences in the rates of multiple medical comorbidities that were consistently more profound in Bedouin children than in Jewish children. Specifically, allergy/hypersensitivity diagnoses were significantly more prevalent in Bedouin cases *v*. Bedouin but no such differences were seen in Jewish children (OR = 2.69; 95% CI 1.52–4.77 *v*. OR = 1.33; 95% CI 0.95–1.84, respectively; Breslow–Day *p*-value = 0.033). Similar ethnic heterogeneity in case–control differences were seen in the rates of cardiovascular diseases (OR = 3.66; 95% CI 1.66–8.07 *v*. OR = 1.28; 95% CI 0.72–2.28 for Bedouin and Jewish children, respectively; Breslow–Day *p*-value = 0.032), GI diseases (OR = 4.42; 95% CI 1.87–10.50 *v*. OR = 1.32; 95% CI 0.74–2.37 for Bedouin and Jewish children, respectively; Breslow–Day *p*-value = 0.019), neurological disorders (OR = 16.08; 95% CI 8.49–30.47 *v*. OR = 5.67, 95% CI 3.58–8.97 for Bedouin and Jewish children, respectively; Breslow–Day *p*-value = 0.009) and psychiatric conditions (OR = 18.57; 95% CI 8.50–40.57 *v*. OR = 3.10; 95% CI 1.88–5.12 for Bedouin and Jewish children, respectively; Breslow–Day *p*-value < 0.001).

### Health services' utilisation patterns

We also compared patterns of health care services' utilisation between children with and without ASD in our sample. As anticipated, there was a higher rate for children with ASD utilizing health services than children without ASD across a variety of health care services (Table 3). Specifically, children with ASD visited their primary-care physician more often, with 0–1 visits per year for 26.8% and >1 visits for 6.5% of children with ASD, compared to 19.7%, and 5.8% for the control children (OR = 1.52; 95% CI 1.20–1.92 and OR = 1.26; 95% CI 0.84–1.91, respectively). Children with ASD were also referred more often to the Emergency Department, with 20.1% of ASD children being referred to the Emergency Department more than once a year compared to only 11.9% of the control children (OR = 2.13; 95% CI 1.57–2.89). Among the children referred to the Emergency Department, the children with ASD were more likely to be admitted to the hospital and to stay longer at the hospital than the control children (OR = 1.28; 95% CI 1.00–1.63 and OR = 2.08; 95% CI 1.59–2.71 for 0–1 and >1 admissions per year, respectively, and OR = 1.44; 95% CI 1.00–2.07 and OR = 1.25; 95% CI 0.80–1.95 for 1–3 and >3 admission days, respectively). While some of these differences were explained by the higher number of comorbidities in children with ASD, some of them remained significant even after adjusting for this factor (Table 3). There were no significant differences between children with and without ASD in the admission rates in the surgical or intensive care wards (Table 3).

**Table 3.** Comparison of healthcare utilisation patterns between children with and without ASD

	ASD *N* = 459	No ASD *N* = 2285	OR^a^ (95% CI)	aOR^b^ (95% CI)
Primary physician visits per year				
0	306 (66.7%)	1702 (74.5%)	Ref	
0–1	123 (26.8%)	451 (19.7%)	**1.52 (1.20–1.92)**	1.20 (0.94–1.53)
>1	30 (6.5%)	132 (5.8%)	1.26 (0.84–1.91)	0.89 (0.71–1.12)
ED referral per year				
0	119 (25.9%)	737 (32.3%)	Ref	
0–1	247 (53.8%)	1278 (55.9%)	1.20 (0.95–1.52)	1.06 (0.83–1.35)
>1	93 (20.1%)	270 (11.9%)	**2.13 (1.57–2.89)**	**1.28 (1.08–1.50)**
Admissions per year				
0	249 (54.2%)	1482 (64.9%)	Ref	
0–1	112 (24.4%)	522 (22.8%)	**1.28 (1.00–1.63)**	1.10 (0.86–1.42)
>1	98 (21.4%)	281 (12.3%)	**2.08 (1.59–2.71)**	**1.28 (1.11–1.47)**
Admission length (days)				
0–1	52 (24.8%)	251 (31.3%)	Ref	
1–3	114 (54.3%)	382 (47.6%)	**1.44 (1.00–2.07)**	1.34 (0.92–1.95)
>3	44 (21.0%)	170 (21.1%)	1.25 (0.80–1.95)	1.08 (0.86–1.36)
Paediatric ward				
Surgical	28 (6.1%)	102 (4.5%)	1.39 (0.90–2.14)	1.04 (0.66–1.65)
Intensive care	9 (2.0%)	33 (1%)	1.37 (0.65–2.87)	0.86 (0.38–1.96)

a Crude odds ratio (OR) and 95% confidence intervals (CI).

b Odds ratio (OR) and 95% confidence intervals (CI) adjusted for the number of comorbidities of a child.

Statistically significant odds ratios (p<0.05) are highlighted in bold font.

## Discussion

The findings of this study contribute to the overall understanding of comorbid conditions and health services' utilisation for children with ASD. This study is, to the best of our knowledge, the most comprehensive of its kind. The study differs from others in the field in that the analyses were not restricted to particular clinical conditions but rather examined a whole plethora of diseases and clinical diagnoses made for children with ASD. The classification systems that we used allowed us to confirm previous conclusions while at the same time deepening the understanding of the specific comorbid conditions in children with ASD.

There is a wide range of evidence indicating that children with ASD are at a higher risk of suffering from other psychiatric or neurological conditions. For example, up to 85% of children with ASD may present symptoms of ADHD (Leitner, 2014) and 8.7–20% of children with ASD also have epilepsy (Kohane *et al*., 2012). In our study, both these conditions were significantly more prevalent in children with ASD than in the control children, but the rates for these conditions in our sample were considerably lower than those previously reported. The relatively young age of the children in our sample (mean = 5.5 ± 1.9 years) may account for the relatively low prevalence of these conditions, which, in many cases, are diagnosed at later ages (Caye *et al*., 2017; Walsh *et al*., 2017).

Children with ASD tend to suffer from non-psychiatric or neurological comorbidities, as depicted in our study and in previous studies as well (Vargason *et al*., 2019). Previous studies assessed higher rates of comorbidity burden within children with ASD, estimating the vast majority of above 90% of children with ASD suffer from a co-occuring medical condition, as depicted by Vargason *et al*. (2019) and Soke *et al*. (2018). Some of these studies were conducted in a hospital or clinic setting, including only children who seeked medical attention. Our study comprised of a cohort including all children diagnosed with ASD who were born in a regional tertiary hospital, some of which had not seeked medical attention at their primary physician and were never reffered to the ER or admitted to the hospital until completion of the study timeline, and therefore show lower rates of comorbidities.

Many children with ASD suffer from GI problems. As shown in other studies, (McElhanon *et al*., 2014), we also found significantly higher rates of GI conditions in children with ASD compared to the control group. However, here too, the prevalence of GI diseases (including inflammatory bowel disease) in the children with ASD was lower than that previously reported (Valicenti-McDermott *et al*., 2006; Kohane *et al*., 2012; Engelchin-Nissan and Shmueli, 2015). We believe that the reason for this difference in prevalence was that the clinical diagnoses used in our study were made in a community care setting and not in a hospital where GI diseases are a common cause of admission of the paediatric population. The reason for the consistent association between ASD and GI conditions has not been clarified definitively and remains open to debate. Some studies have demonstrated that children with ASD have a different intestinal microbiome, which might be associated with their GI conditions (Finegold, 2011). Other studies have suggested that the particular nutritional behaviours of many children with ASD could also contribute to their intestinal problems or, conversely, could be an outcome of these problems (Valicenti-McDermott *et al*., 2006; Engelchin-Nissan and Shmueli, 2015). Finally, GI inflammation has also been suggested as a possible mechanism that underlies various neurological and psychiatric conditions, including ASD (Brierley and Linden, 2014). The higher prevalence of allergies and other hypersensitive conditions found in our study may also support this hypothesis, similarly to a recent study that found that children with food, respiratory and skin allergies were more likely to have ASD (Xu *et al*., 2018). These allergies are common conditions of immunological dysfunction in children, and all of them, especially food allergies, are associated with a higher burden of ASD. Taken together, the above findings suggest that the immune system may play a role in the underlying aetiology of ASD.

It is well known that many children with ASD have exceptional and unusual sensory perceptions. In our study, we found a higher prevalence of visual and auditory impairments in children with ASD. Indeed, the associations of both these sensory impairments with ASD were also observed in other studies (Doshi-Velez *et al*., 2014; Engelchin-Nissan and Shmueli, 2015; Kiani *et al*., 2019). Yet, controversy still exists in the literature regarding the prevalence of hearing impairments in individuals with ASD (Beers *et al*., 2014). It is possible that a disturbance in the perception of hearing is linked to a disturbance in the development of normal communication and social skills, as presented in ASD. Similarly, visual impairments could also contribute to communication deficiencies, especially those related to eye contact. Nevertheless, further studies are needed to determine whether the association between visual and auditory conditions and ASD is due to mutual neurological impairments or due to the communication difficulties that such conditions impose upon these children.

We also found higher rates of congenital conditions in the cardiovascular, dermatological, endocrinological and orthopaedic systems. Since children with known genetic disorders were excluded from the analysis, these differences might reflect exposure to other (genetic and non-genetic) prenatal risk factors associated with ASD and other congenital abnormalities. Thus, these associations suggest that common biological mechanisms may underlie both ASD and other congenital conditions. An alternative explanation could be that the manifestation of these diseases predisposes a susceptibility to ASD or *vice versa* (Bean Jaworski *et al*., 2017). Overall, the greater tendency of children with ASD to manifest other comorbidities across a wide range of pathophysiological and anatomical/systemic medical classes implies that ASD is not solely a developmental disorder defined by communication, behavioural and social impairments, but rather a multisystem condition involving additional comorbid conditions. It is self-evident that such a comorbidity burden must have a significant impact on the overall health and wellbeing of children with ASD and their families.

We found more profound case–control differences in medical comorbidities in Bedouin children than in Jewish children. This indicates that the overall medical burden in Bedouin children with ASD is larger than in Jewish children. This finding is in line with a previous report from our group that found that Bedouin children tend to be diagnosed with more severe forms of ASD than Jewish children (Levaot *et al*., 2019), and this difference likely to be related to the possible underdiagnoses of milder ASD cases in this population. Such under-representation of ASD cases with milder symptoms and possibly lower rates of other medical comorbidities may lead to slight overestimation of the case–control differences in medical comorbidities that were found in this study.

We found that children with ASD tend to be referred – and subsequently admitted – to the hospital more frequently than children without ASD, regardless of the existing comorbidity burden. It is not likely that this difference is related to differences in the severity of the problem requiring referral to the Emergency Department, because no significant differences were found in the length of hospitalisation and the type of paediatric ward between the study groups. Therefore, we think that the more frequent hospital referrals and admissions of children with ASD are due to the greater difficulty of both parents and physicians to understand the physiological complaints of these children. In addition, the higher alertness of parents of children with ASD to changes in their medical condition and the greater challenge in the handling of such illnesses at home may also contribute to these differences in healthcare utilisation. Overcoming these challenges and thereby reducing the excessive use of healthcare services by these children is a public health priority, *inter alia* because it will *per se* reduce the attendant healthcare expenditure (Liptak *et al*., 2006).

The major strengths of this study lie in the relatively large and comprehensive medical data set that was obtained from a single public health provider and the relatively robust diagnoses of both the outcome variable (i.e. ASD) and the chronic comorbidities. However, there are also some study limitations: First, although the medical databases that we used document all diagnoses and public healthcare utilisation of the children in the study, we might have missed any medical care of these children that was handled in the private medical sector. However, since there is public health insurance for all Israeli citizens, the use of private medical insurance is extremely rare for children, especially in southern Israel (Engelchin-Nissan and Shmueli, 2015). Second, despite the relatively large sample size of this study, it did not have the statistical power to explore differences in rare chronic conditions between the study groups. Third, we did not have data about the clinical complaints of the children who were referred to the Emergency Department. Yet, as noted above, we did have data for the length of hospitalisation and the type of ward, which might be good proxies for the illness severity of these children.

## Conclusions

The higher comorbid burden and health care service utilisation observed for children with ASD in this study highlight the greater health-related needs of these children. Increasing the awareness of healthcare professionals of these needs, and developing tools to manage difficulties associated with them, will improve the quality of healthcare provided to these children, and reduce the attendant medical, emotional and financial burdens.

## Supplementary material

For supplementary material accompanying this paper visit http://dx.doi.org/10.1017/S2045796020000050. click here to view supplementary material

## Data

The data supporting the findings of this study are available upon request.
