# Supplementary material for: Comorbidity and health services' usage in children with autism spectrum disorder: a nested case–control study
Source: Epidemiol Psychiatr Sci. 2020 Jan 28;29:e95. doi: 10.1017/S2045796020000050 (PMC7214718; doi:10.1017/S2045796020000050)
Supplement: Supplementary file 1 [file S2045796020000050sup001.docx]

**Supplementary Tables**

**Table S1: Classification of diagnoses via pathophysiological classification**

| PATHOPHYSIOLOGICAL CLASSIFICATION | LIST OF DIAGNOSES | ICD-9 code |
| --- | --- | --- |
| ALLERGY/HYPERSENSITIVITY | ADENOID HYPERTROPHY | 474.12 |
|  | ADENOIDECTOMY WITHOUT TONSILLECTOMY | 28.2-28.3 |
|  | ALLERGIC CONJUNCTIVITIS | 372.14 |
|  | ALLERGY | 995.3 |
|  | ALLERGY BEE STING (ANAPHYLATIC SHOCK) | 995.3 |
|  | ALLERGY DRUG | 995.27 |
|  | ALLERGY FOOD | 995.3 |
|  | ANAPHYLACTIC SHOCK | 995.0 |
|  | ANGIOEDEMA | 995.1 |
|  | ASTHMA | 493.9 |
|  | ATOPIC DERMATITIS | 691.8 |
|  | ATOPIC DERMATITIS/ECZEMA | 691.8 |
|  | BRONCHIAL ASTHMA | 493.92 |
|  | CONJUNCTIVITIS | 372.30 |
|  | DERMATOGRAPHIA | 708.3 |
|  | DRUG ALLERGIC REACTION | 995.27 |
|  | HYPERTROPHIC TONSILS & ADENOIDS | 474.12 |
|  | MILK ALLERGY | 995.3 |
|  | POLYPS NASAL | 471.0 |
|  | RHINITIS ALLERGIC | 477.9 |
|  | RHINITIS CHRONIC | 472.0 |
|  | RHINITIS VASOMOTOR | 472.0 |
|  | RHINORRHEA | 349.81 |
|  | SYSTEMIC FOOD DERMATITIS | 692.9 |
|  | TONSIL AND ADENOID HYPERTROPHY | 472.0 |
|  | TONSIL HYPERTROPHY ALONE | 474.10 |
|  | TONSILLECTOMY WITH ADENOIDECTOMY | 28.00 |
|  | TONSILLECTOMY WITHOUT ADENOIDECTOMY | 28.00 |
|  | TONSILS WITH ADENOIDS | 474.10 |
|  | URTICARIA | 708.9 |
|  | URTICARIA ALLERGIC | 708.00 |
|  | URTICARIA CHRONIC | 708.9 |
| ANEMIA | ANEMIA HYPOCHROMIC | 280.9 |
|  | ANEMIA MICROCYTIC | 280.9 |
|  | ANEMIA NUTRITIONAL | 281.8 |
|  | ANEMIA TO G6PD DEFICIENCY | 282.2 |
|  | HYPOCHROMIC ANEMIA | 280.9 |
|  | IRON DEFICIENCY ANEMIA | 280.9 |
| CONGENITAL | ANKYLOGLOSSIA | 750.0 |
|  | ARACHNOID CYST | 349.2 |
|  | ASYMMETRY HEAD | 754.0 |
|  | ATRESIA CHOANAL | 748.0 |
|  | ATRIAL SEPTAL DEFECT | 745.5 |
|  | ATROPHY OF TESTIS | 608.3 |
|  | BICUSPID AORTIC VALVE | 746.4 |
|  | BRONCHOPULMONARY DYSPLASIA | 770.7 |
|  | CATARACT NUCLEAR CONGENITAL | 366.9 |
|  | CEREBRAL PALSY | 343.9 |
|  | CLEFT LIP | 749.20 |
|  | CLEFT PALATE | 749.20 |
|  | CLEFT PALATE UNILATERAL INCOMPLETE | 749.20 |
|  | CLEFT PALATE+CLEFT LIP | 749.20 |
|  | COLOBOMA OF IRIS CONGENITAL | 743.46 |
|  | COLOBOMA OPTIC DISC | 377.23 |
|  | CRANIOSYNOSTOSIS | 756.0 |
|  | CROUZON SYNDROME | 756.0 |
|  | CRYPTORCHISM | 752.51 |
|  | DACRYOSTENOSIS | 375.55 |
|  | DEPRIVATION AMBLYOPIA | 368.00 |
|  | DEVIATED NASAL SEPTUM | 470.00 |
|  | DISLOCATION HIP CONGENITAL | 754.30 |
|  | DIVERTICULUM OF BLADDER | 596.3 |
|  | DUANES SYNDROME | 378.71 |
|  | DYSPLASIA ACETABULAR | 754.30 |
|  | DYSPLASIA HIP | 754.30 |
|  | DYSPLASIA HIP CONGENITAL | 754.30 |
|  | ECTOPIC KIDNEY | 753.3 |
|  | ECTOPIC KYDNEY | 753.3 |
|  | FISTULA ANORECTAL | 565.1 |
|  | FUSION OF KIDNEY | 753.3 |
|  | GENU VARUS - BOWLEGS CONGENITAL | 736.42 |
|  | GLAUCOMA CONGENITAL | 365.14 |
|  | HYDROCEPHALUS CONGENITAL | 742.3 |
|  | HYPOSPADIA | 752.61 |
|  | HYPOSPADIAS | 752.61 |
|  | HYPOTHYROIDISM CONGENITAL | 243.00 |
|  | IGA IMMUNODEFICIENCY | 279.01 |
|  | IMMUNODEFIENCY WITH T-CELL DEFECT | 279.10 |
|  | IMPERFORATE ANUS | 751.2 |
|  | LARYNGOMALACIA/CONGENITAL | 748.3 |
|  | MEATAL STENOSIS | 598.9 |
|  | METATARSUS ADDUCTUS | 754.53 |
|  | METATARSUS ADDUCTUS CONGENITAL | 754.53 |
|  | MICROCEPHALY | 742.1 |
|  | MICROCHEILIA | 744.81 |
|  | MICROSTOMIA | 744.81 |
|  | NEVUS CONGENITAL | 216.9 |
|  | NYSTAGMUS CONGENITAL | 379.50 |
|  | OSTEOGENESIS IMPERFECTA | 756.51 |
|  | PATENT DUCTUS ARTERIOSUS | 747.0 |
|  | PATENT FORAM OVALE | 745.5 |
|  | PATENT FORAMEN OVALE | 745.5 |
|  | PECTUS EXCAVATUM | 754.81 |
|  | PECTUS EXCAVATUM CONGENITAL | 754.81 |
|  | PIGEON-TOE | 735.8 |
|  | PTOSIS CONGENITAL | 743.61 |
|  | PULMONARY STENOSIS CONGENITAL | 746.02 |
|  | PULMONARY VALVE DISEASE | 424.3 |
|  | SCLERA CONGENITAL ANOMALIES | 743.49 |
|  | SHORT LEG CONGENITAL | 755.30 |
|  | STENOSIS PULMONARY CONGENITAL | 746.02 |
|  | STENOSIS TEARDUCT CONGENITAL | 375.55 |
|  | STERNOCLEIDOMASTOID CONGENITAL DEFORMITY | 754.1 |
|  | STRABISMUS | 378.73 |
|  | STRIDOR CONGENITAL | 786.1 |
|  | SYNDACTYLY | 755.11 |
|  | SYNDACTYLY TOES WITHOUT BONE FUSION | 755.14 |
|  | TONGUE TIE | 750.0 |
|  | TORTICOLLIS CONGENITAL | 723.5 |
|  | UNDESC.TESTICLE/CRYPTORCHISM | 752.51 |
|  | UNDESCENDED TESTIS | 752.51 |
|  | UNRETRACTABLE FORESKIN | 605.00 |
|  | VENTRICULAR SEPTAL DEFECT | 745.4 |
|  | VITILIGO | 709.01 |
| DEVELOPMENTAL | DELAY IN DEVELOPMENT PHYSIOLOGICAL | 315.9 |
|  | DELAY IN MENTAL DEVELOPMENT PHYSIOLOGICAL | 315.9 |
|  | DELAYED MOTOR MILESTONES | 315.9 |
|  | DEVELOPMENT DELAY | 315.9 |
|  | DEVELOPMENT LACK OF - PHYSIOLOGIC | 315.9 |
|  | DEVELOPMENT SLOW | 315.9 |
|  | DEVELOPMENTAL DELAY | 315.9 |
|  | DEVELOPMENTAL LEARNING DIFFICULTIES | 315.2 |
|  | DYSPHASIA | 784.3 |
|  | GLOBAL DEVELOPMENT DELAY | 315.9 |
|  | INTELLECTUAL DISABILITIES | 317.00-319.00 |
|  | RETARDATION MENTAL | 319.00 |
|  | SLOW DEVELOPMENT | 315.9 |
|  | UNDERDEVELOPMENT PHYSIOLOGIC | 783.40 |
| DEVELOPMENTAL-SPEECH/LANGUAGE | DEVELOPMENTAL LANGUAGE DISORDER | 315.39 |
|  | DEVELOPMENTAL SPEECH OR LANGUAGE DISORDER | 315.39 |
|  | DISTURBANCE OF SPEECH | 315.39 |
|  | SPEECH DEFECT TRAINING | 315.39 |
|  | SPEECH DISTURBANCE | 315.39 |
|  | SPEECH OR LANGUAGE DISORDERS | 315.39 |
| GENETIC | ALBINISM | 270.2 |
|  | ANGELMANS SYNDROME | 759.89 |
|  | FAMILIAL MEDITERRANEAN FEVER | 277.31 |
|  | GLUCOSE-6-PHOSPHATE DEFICIENCY | 282.2 |
|  | GLUCOSE-6-PHOSPHATE DEHYDROGENASE DEFICIENCY | 282.2 |
|  | GLYCOGEN STORAGE DISEASE | 271.0 |
|  | KERATODERMA | 701.1 |
|  | PFAPA (PERIODIC FEVER APHTHOUS STOMATITIS PHARYNGITIS & A | 277.31 |
|  | PFAPA (PERIODIC FEVER APHTHOUS STOMATITIS PHARYNGITIS & ADENOPATHY) | 277.31 |
|  | PHENYLKETONURIA | 270.1 |
|  | PHENYLKETONURIA PKU | 270.1 |
|  | STURGE WEBER SYNDROME | 759.6 |
|  | THALASSEMIA | 282.40 |
|  | THALASSEMIA MINOR | 282.46 |
|  | VON GIERKE`S DISEASE | 271.0 |
| HEARING IMPAIRMENT | CONDUCTIVE HEARING LOSS | 389.00 |
|  | DEAFNESS | 389.9 |
|  | DEAFNESS CONDUCTIVE TYMPANIC MEMRANE | 389.00 |
|  | DEAFNESS SENSORINEURAL SENSORY | 389.10 |
|  | DEAFNESS/PARTIAL OR COMPLETE | 389.00 |
|  | HEARING COMPLAINTS | 389.8 |
|  | HEARING EXAMINATION NOT OTHERWISE SPECIFIED | 389.8 |
|  | LOSS OF HEARING | 389.00 |
|  | SENSORINEURAL HEARING LOSS | 389.00 |
| INFECTIOUS | ARTHRITIS INFECTIOUS | 711.0 |
|  | BRONCHIOLITIS ACUTE | 466.00 |
|  | BRONCHOPNEUMONIA | 485.00 |
|  | CONJUNCTIVITIS VIRAL | 372.30 |
|  | OSTEOMYELITIS ACUTE | 730.0 |
|  | CYTOMEGALIC INCLUSION DISEASE | 078.5 |
|  | DACRYOCYSTITIS | 375.30 |
|  | DYSENTERY DIARRHEA | 004.9 |
|  | ECZEMATOID | 692.9 |
|  | GIARDIA LAMBLIASIS | 007.1 |
|  | GIARDIAL COLITIS | 558.9 |
|  | HEPATITIS C | 070.54 |
|  | HORDEOLUM EXTERNUM | 373.11 |
|  | INFECTION URINARY TRACT NOS | 599.0 |
|  | LISTERIOSIS | 027.0 |
|  | MENINGITIS ASEPTIC VIRAL | 047.9 |
|  | MENINGITIS E. COLI | 322.9 |
|  | MENINGITIS PNEUMOCOCCAL | 320.1 |
|  | MONONUCLEOSIS INFECTIOUS | 075.00 |
|  | OTITIS EXUDATIVE CHRONIC | 381.10 |
|  | OTITIS MEDIA ACUTE | 382.0 |
|  | OTITIS MEDIA ACUTE SEROUS | 381.0 |
|  | OTITIS MEDIA CHRONIC MUCOID GLUE | 381.1 |
|  | OTITIS MEDIA CHRONIC SEROUS | 381.1 |
|  | OTITIS MEDIA CHRONIC SUPPURATIVE | 382.3 |
|  | OTITIS MEDIA CHRONIC WITH EFFUSION | 381.1 |
|  | OTITIS MEDIA NON-SUPPURATIVE ACUTE | 381.4 |
|  | OTITIS SEROUS CHRONIC | 381.1 |
|  | OTORRHEA | 388.60 |
|  | OTORRHEA UNSPECIFIED | 388.60 |
|  | OXYURIASIS | 127.4 |
|  | PERIANAL ABSCESS | 566.00 |
|  | PERITONSILLAR ABSCESS | 475.00 |
|  | PNEUMONIA | 482.9 |
|  | PNEUMONIA HEMOPHILUS INFLUENZA | 482.2 |
|  | SEPSIS NEONATAL | 771.81 |
|  | THREADWORMS | 127.4 |
|  | THRUSH MOUTH | 112.0 |
|  | UPPER RESPIRATORY TRACT INFECTION | 465.9 |
|  | URI | 465.9 |
|  | URINARY TRACT INFECTION | 599.0 |
|  | URINARY TRACT INFECTION SITE UNSPECIFIED | 599.0 |
|  | VARICELLA | 052.0 |
| NEUROLOGICAL-CONVULSION RELATED | COMPLEX FEBRILE SEIZURES | 780.31 |
|  | CONTINUOUS SPIKE WAVE DURING SLOW WAVE SLEEP CSWS | 794.02 |
|  | CONVULSIONS | 780.3 |
|  | CONVULSIONS FEBRILE | 780.31 |
|  | CONVULSIONS NEWBORN | 780.3 |
|  | CONVULSIONS NOS | 780.39 |
|  | CONVULSIONS/SEIZURES | 780.3 |
|  | EPILEPSY | 345.9 |
|  | GENERALIZED EPILEPSY WITH FEBRILE SEIZURES PLUS | 345.10 |
|  | INFANTIL SPASMS | 345.6 |
|  | LENNOX GASTAUT SYNDROME | 345.01 |
|  | SEVERE MYOCLONIC EPILEPSY OF INFANCY - DRAVET SYNDROME | 345.11 |
|  | BMI PEDIATRIC OBESE GREATER THAN OR EQUAL TO 97TH PERCENT | 278.0 |
|  | BMI PEDIATRIC OVERWEIGHT 97TH < BMI < 99.9TH PERCENTILE FOR AGE | 278.0 |
|  | BMI PEDIATRIC RISK FOR OBESITY 85TH < BMI <= 97TH PERCENTILE FOR AGE | 278.0 |
|  | BMI PEDIATRIC RISK FOR OBESITY 85TH < BMI > 97TH PERCENTI | 278.0 |
|  | BMI PEDIATRICS OBESE GREATHER OR EQUAL TO 95TH PERCENTIL | 278.0 |
|  | OBESITY | 278.0 |
|  | OBESITY (BMI >30) | 278.0 |
|  | OVERWEIGHT (BMI < 30) | 278.02 |
| SLEEP APNEA | APNEA - SLEEP | 786.03 |
|  | OBSTRUCTIVE SLEEP APNEA | 786.03 |
|  | SLEEP APNEA | 786.03 |
| TRAUMA | ACCIDENT/INJURY; NOS | E928.9 |
|  | BLACK EYE TRAUMATIC | E928.9 |
|  | CEPHALHEMATOMA BIRTH INJURY | 920.0 |
|  | CONTUSION | 924.9 |
|  | CONTUSION EYE | 921.3 |
|  | FALL | E880-E888 |
|  | FALLS RECURRENT | V15.88 |
|  | FRACTURE ANKLE | 824.8 |
|  | FRACTURE CLAVICLE | 810.0 |
|  | FRACTURE CLAVICLE CLOSED | 810.00 |
|  | FRACTURE FACIAL BONES | 802.0 |
|  | FRACTURE FEMUR PERTROCHANTERIC CLOSED | 820.22 |
|  | FRACTURE HUMERUS SUPRACONDYLAR CLOSED | 812.41 |
|  | FRACTURE METACARPALS CLOSED | 815.00 |
|  | FRACTURE PHALANX/PHALANGES CLOSED | 816.00 |
|  | FRACTURE RADIUS | 813.0 |
|  | FRACTURE RADIUS NECK CLOSED | 813.06 |
|  | FRACTURE SKULL | 803.00 |
|  | FRACTURE TIBIA AND FIBULA SHAFT CLOSED | 823.2 |
|  | FRACTURE TIBIA WITH FIBULA CLOSED | 823.22 |
|  | FRACTURE ULNA SHAFT ALONE CLOSED | 813.82 |
|  | HEAD INJURY | 959.01 |
|  | HEMATOMA | 998.12 |
|  | INTRACRANIAL INJURY WITH SKULL FRACTURE | 803.1 |
|  | MOTOR VEHICLE ACCIDENT | E810-E819 |
|  | SPLEEN INJURY | 865.0 |
| VISUAL IMPAIRMENT | ASTIGMATISM | 367.20 |
|  | ASTIGMATISM REGULAR | 367.20 |
|  | CHOROIDEREMIA | 363.55 |
|  | HYPERMETROPIA | 367.0 |
|  | HYPEROPIA | 367.0 |
|  | MYOPIA | 367.1 |
|  | SHORT SIGHTEDNESS | 367.1 |

**Table S2: Classification of diagnoses via anatomical/systemic classification**

| ANATOMICAL/SYSTEMIC CLASSIFICATION | LIST OF DIAGNOSES | ICD-9 code |
| --- | --- | --- |
| ABDOMINAL WALL | HERNIA FEMORAL | 553.0 |
|  | HERNIA INGUINAL UNILATERAL | 550.90 |
|  | HERNIA INGUINAL UNILATERAL RECURRENT | 550.90 |
|  | HERNIA UMBILICAL | 552.0 |
|  | INGUINAL HERNIA | 550.0 |
|  | UMBILICAL HERNIA | 552.0 |
| ADENOID/TONSILS | ADENOID HYPERTROPHY | 474.12 |
|  | ADENOIDECTOMY WITHOUT TONSILLECTOMY | 28.2-28.3 |
|  | HYPERTROPHIC TONSILS & ADENOIDS | 474.12 |
|  | TONSIL AND ADENOID HYPERTROPHY | 472.0 |
|  | TONSIL HYPERTROPHY ALONE | 474.10 |
|  | TONSILLECTOMY WITH ADENOIDECTOMY | 28.00 |
|  | TONSILLECTOMY WITHOUT ADENOIDECTOMY | 28.00 |
|  | TONSILLITIS ACUTE | 463.0 |
|  | TONSILS WITH ADENOIDS | 474.10 |
| AURICULAR | HEARING COMPLAINTS | 389.9 |
|  | HEARING EXAMINATION NOT OTHERWISE SPECIFIED | V72.19 |
|  | SEROUS OTITIS MEDIA | 381.01 |
| CARDIOVASCULAR | ABNORMAL CARDIOVASCULAR FUNCTION STUDY | 794.30 |
|  | ATRIAL SEPTAL DEFECT | 745.5 |
|  | ATRIOVENTRICULAR BLOCK COMPLETE | 426.0 |
|  | BICUSPID AORTIC VALVE | 424.1 |
|  | CARDIOMEGALY | 429.3 |
|  | HEART MURMUR; NOS | 785.2 |
|  | INNOCENT HEART MURMUR | 785.2 |
|  | ISCHEMIC HEART DISEASE SUBACUTE | 414.9 |
|  | MURMUR HEART FUNCTIONAL INNOCENT | 785.2 |
|  | MURMUR HEART INNOCENT | 785.2 |
|  | MURMUR HEART SYSTOLIC | 785.2 |
|  | PATENT FORAM OVALE | 745.5 |
|  | PATENT FORAMEN OVALE | 745.5 |
|  | PULMONARY HYPERTENSION | 416.0 |
|  | PULMONARY HYPERTENSION PRIMARY | 416.0 |
|  | PULMONARY HYPERTENSION SECONDARY | 416.0 |
|  | PULMONARY STENOSIS CONGENITAL | 747.31 |
|  | PULMONARY VALVE DISEASE | 424.3 |
|  | STENOSIS PULMONARY CONGENITAL | 747.31 |
|  | SUPRAVENTRICULAR TACHYCARDIA | 427.0 |
|  | SYSTOLIC MURMUR | 785.2 |
|  | VENTRICULAR SEPTAL DEFECT | 745.4 |
| DERMATOLOGICAL | ALBINISM | 757.33 |
|  | ANGIOMA | 228.00 |
|  | CAFE AU LAIT SPOTS | 709.09 |
|  | CYST EPIDERMAL | 706.2 |
|  | DERMATOGRAPHIA | 708.3 |
|  | KERATODERMA | 701.1 |
|  | NEVUS BLUE | 216.9 |
|  | NEVUS CONGENITAL | 216.9 |
|  | NEVUS EPIDERMAL | 216.9 |
|  | NEVUS SEBACEOUS | 216.9 |
|  | PAPILLOMA SKIN | 079.4 |
|  | PERIORAL DERMATITIS | 691.0 |
|  | SEBACEOUS CYST | 706.2 |
|  | SEBORRHEA | 706.3 |
|  | SEBORRHEIC DERMATITIS | 706.3 |
|  | SEBORRHOEIC ECZEMA | 706.3 |
|  | SKIN TAG | 701.19 |
|  | SYSTEMIC FOOD DERMATITIS | 693.1 |
|  | VITILIGO | 709.01 |
| ENDOCRINOLOGICAL | BMI PEDIATRIC OBESE GREATER THAN OR EQUAL TO 97TH PERCENT | 278.0 |
|  | BMI PEDIATRIC OVERWEIGHT 97TH < BMI < 99.9TH PERCENTILE FOR AGE | 278.0 |
|  | BMI PEDIATRIC RISK FOR OBESITY 85TH < BMI <= 97TH PERCENTILE FOR AGE | 278.0 |
|  | BMI PEDIATRIC RISK FOR OBESITY 85TH < BMI > 97TH PERCENTI | 278.0 |
|  | BMI PEDIATRIC UNDERWEIGHT LESS THAN 3RD PERCENTILE FOR AG | 783.22 |
|  | DIABETES INSIPIDUS | 253.5 |
|  | DIABETES MELLITUS | 250 |
|  | DIABETES MELLITUS JUVENILE ONSET | 250 |
|  | EATING DISORDER | 307.50 |
|  | GLUCOSE-6-PHOSPHATE DEFICIENCY | 277.6 |
|  | GLUCOSE-6-PHOSPHATE DEHYDROGENASE DEFICIENCY | 277.6 |
|  | GLYCOGEN STORAGE DISEASE | 271.0 |
|  | GYNECOMASTIA | 611.1 |
|  | HIRSUTISM | 704.1 |
|  | HIRSUTISM ACQUIRED | 704.1 |
|  | HORMONE REPLACEMENT THERAPY | V07.4 |
|  | HYPERBILIRUBINEMIA | 277.4 |
|  | HYPERBILIRUBINEMIA NEWBORN | 774.6 |
|  | HYPERKERATOSIS | 102.3 |
|  | HYPERTRICHOSIS | 374.54 |
|  | HYPOGLYCEMIA | 251.2 |
|  | HYPOGLYCEMIA UNSPECIFIED | 251.2 |
|  | HYPOTHYROIDISM | 243.0 |
|  | HYPOTHYROIDISM ACQUIRED | 244.0 |
|  | HYPOTHYROIDISM CONGENITAL | 243.0 |
|  | HYPOTHYROIDISM/MYXEDEMA | 244.9 |
|  | MODY MATURE ONSET DIABETES IN YOUNG | 250.00 |
|  | MYXEDEMA CIRCUMSCRIBED | 244.9 |
|  | MYXEDEMA PRIMARY NOT SPECIFIED | 244.9 |
|  | OBESITY | 278.0 |
|  | OVERDEVELOPMENT BREAST | 259.1 |
|  | OVERWEIGHT (BMI < 30) | 278.0 |
|  | PANHYPOPITUITARISM | 253.2 |
|  | PHENYLKETONURIA | 270.1 |
|  | PHENYLKETONURIA PKU | 270.1 |
|  | PHYSIOLOGICAL JAUNDICE OF NEWBORN | 774.6 |
|  | SEXUAL PRECOCITY | 259.1 |
|  | SHORT STATURE | 783.43 |
|  | VITAMIN D DEFICIENCY | 268.9 |
|  | VON GIERKE`S DISEASE | 271.0 |
| GASTROINTESTINAL | APPENDECTOMY | 540.9 |
|  | APPENDICITIS | 540.9 |
|  | APPENDICITIS WITH PERITONEAL ABSCESS | 540.1 |
|  | BLOOD IN STOOLS | 578.1 |
|  | CELIAC DISEASE | 579.0 |
|  | CHOLELITHIASIS | 574.0 |
|  | CONSTIPATION | 564.00 |
|  | CROHNS DISEASE | 555.0 |
|  | DIARRHEA | 787.91 |
|  | ESOPHAGEAL REFLUX | 530.81 |
|  | ESOPHAGUS ACHALASIA | 530.0 |
|  | FECAL IMPACTION | 560.32 |
|  | HEPATOMEGALY | 789.1 |
|  | IMPERFORATE ANUS | 751.2 |
|  | INTUSSUSCEPTION | 560.0 |
|  | LACTOSE INTOLERANCE | 271.3 |
|  | MELENA | 578.1 |
|  | MILK ALLERGY | V15.02 |
|  | PILONIDAL FISTULA | 685.1 |
|  | REFLUX | 530.81 |
|  | REFLUX ESOPHAGEAL | 530.81 |
|  | REGURGITATION FOOD | 307.53 |
|  | SPLENOMEGALY | 789.2 |
|  | STEATORRHEA | 579.4 |
|  | VOMITING - EMESIS | 787.0 |
| HEMATOLOGICAL | ANEMIA HYPOCHROMIC | 280.9 |
|  | ANEMIA MICROCYTIC | 280.9 |
|  | ANEMIA NUTRITIONAL | 281.9 |
|  | ANEMIA TO G6PD DEFICIENCY | 282.2 |
|  | HEMATOMA | 729.92 |
|  | HEMOPHILIA | 286.52 |
|  | HEMOPHILIA FACTOR VIII DISORDER | 286.0 |
|  | HYPOCHROMIC ANEMIA | 280.9 |
|  | IRON DEFICIENCY | 280.9 |
|  | IRON DEFICIENCY ANEMIA | 280.9 |
|  | LEUKOPENIA | 288.50 |
|  | NEUTROPENIA | 288.00 |
|  | PURPURA SENILE | 287.2 |
|  | THALASSEMIA | 282.40 |
|  | THALASSEMIA MINOR | 282.46 |
|  | THROMBOCYTOPENIA | 287.5 |
| NEUROLOGICAL | ARACHNOID CYST | 348.0 |
|  | ATAXIA | 334.3 |
|  | CEREBRAL PALSY | 343.0 |
|  | CEREBROVASCULAR ACCIDENT - CVA | 434.0 |
|  | COMPLEX FEBRILE SEIZURES | 780.32 |
|  | CONTINUOUS SPIKE WAVE DURING SLOW WAVE SLEEP CSWS | 794.02 |
|  | CONVULSIONS | 780.3 |
|  | CONVULSIONS FEBRILE | 780.31 |
|  | CONVULSIONS NEWBORN | 780.3 |
|  | CONVULSIONS NOS | 780.39 |
|  | CONVULSIONS/SEIZURES | 780.3 |
|  | DELAY IN DEVELOPMENT PHYSIOLOGICAL | 315.9 |
|  | DELAY IN MENTAL DEVELOPMENT PHYSIOLOGICAL | 315.9 |
|  | DELAYED MOTOR MILESTONES | 315.9 |
|  | DELAYED SPEECH | 315.39 |
|  | DEVELOPMENT DELAY | 315.9 |
|  | DEVELOPMENT LACK OF - PHYSIOLOGIC | 315.9 |
|  | DEVELOPMENT SLOW | 315.9 |
|  | DEVELOPMENTAL DELAY | 315.9 |
|  | DEVELOPMENTAL LANGUAGE DISORDER | 315.39 |
|  | DEVELOPMENTAL LEARNING DIFFICULTIES | 315.2 |
|  | DEVELOPMENTAL SPEECH OR LANGUAGE DISORDER | 315.39 |
|  | DISTURBANCE OF SPEECH | 315.39 |
|  | EPILEPSY | 345.9 |
|  | GENERALIZED EPILEPSY WITH FEBRILE SEIZURES PLUS | 345.10 |
|  | GLOBAL DEVELOPMENT DELAY | 315.9 |
|  | HEADACHE | 784.0 |
|  | HYDROCEPHALUS | 742.3 |
|  | HYDROCEPHALUS COMMUNICATING | 742.3 |
|  | HYDROCEPHALUS CONGENITAL | 742.3 |
|  | INFANTIL SPASMS | 345.6 |
|  | LENNOX GASTAUT SYNDROME | [345.80](http://www.icd9data.com/2015/Volume1/320-389/340-349/345/345.80.htm?__hstc=93424706.57b26b0bc9f5f38ca9481aea332a0051.1573323102131.1573895414725.1573901664347.3&__hssc=93424706.2.1573901664347&__hsfp=2985402543) |
|  | MACROCEPHALY | 756.0 |
|  | MICROCEPHALY | 756.0 |
|  | SEIZURE FEBRILE | 780.31 |
|  | SEVERE MYOCLONIC EPILEPSY OF INFANCY - DRAVET SYNDROME | 345.11 |
|  | SIMPLE FEBRILE SEIZURES | 780.31 |
|  | TREMOR NOS | 781.0 |
| NEUROLOGICAL-OPHTALMOLOGICAL | AMBLYOPIA | 368.00 |
|  | HYPERPHORIA ALTERNATING | 378.45 |
|  | NYSTAGMUS CONGENITAL | 379.50 |
|  | SACCADIC EYE MOVEMENTS DEFICIENCY | 379.57 |
|  | STRABISMUS | 378.73 |
| NEUROMUSCULAR | HYPOTONIA | 781.99 |
|  | MUSCULAR DYSTROPHY | 359.0 |
|  | MYOPATHY | 359.9 |
|  | SPASTIC DIPLEGIA | 342.10 |
| OPHTALMOLOGICAL | ANISOCORIA | 379.41 |
|  | ASTIGMATISM | 367.20 |
|  | CATARACT NUCLEAR CONGENITAL | 743.30 |
|  | CHOROIDEREMIA | 363.55 |
|  | COLOBOMA OF IRIS CONGENITAL | 743.46 |
|  | COLOBOMA OPTIC DISC | 377.23 |
|  | DEPRIVATION AMBLYOPIA | 368.00 |
|  | DUANES SYNDROME | 378.71 |
|  | EPIPHORA EXCESS LACRIMATION | 375.21 |
|  | EPIPHORA INSUFFICIENT DRAINAGE | 375.22 |
|  | ESOTROPIA ALTERNATING | 378.00 |
|  | ESOTROPIA NOT SPECIFIED | 378.00 |
|  | EXOPHORIA | 378.42 |
|  | EXOTROPIA | 378.10 |
|  | EXOTROPIA UNSPECIFIED | 378.10 |
|  | GLAUCOMA | 365.0 |
|  | GLAUCOMA CONGENITAL | 365.14 |
|  | HYDROPHTHALMOS | 743.2 |
|  | HYPERMETROPIA | 367.0 |
|  | HYPEROPIA | 367.0 |
|  | MYOPIA | 367.1 |
|  | PTOSIS CONGENITAL | 743.61 |
|  | RETINITIS PIGMENTOSA | 362.74 |
|  | SCLERA CONGENITAL ANOMALIES | 743.47 |
|  | SHORT SIGHTEDNESS | 367.1 |
|  | STENOSIS TEARDUCT CONGENITAL | 375.56 |
|  | TEAR DUCT OBSTRUCTION | 375.55 |
|  | UVEITIS UNSPECIFIED | 364.3 |
| ORTHOPEDIC | ACETABULAR DYSPLASIA | 754.30 |
|  | BOW LEGS ACQUIRED | 736.42 |
|  | BOWLEG ACQUIRED | 736.42 |
|  | CLUBFOOT | 754.51 |
|  | DEFORMITY FEMUR ACQUIRED | 736.81 |
|  | DISLOCATION HIP CONGENITAL | 754.30 |
|  | DYSPLASIA ACETABULAR | 754.30 |
|  | DYSPLASIA HIP | 754.30 |
|  | DYSPLASIA HIP CONGENITAL | 754.30 |
|  | EOSINOPHILIC GRANULOMA | 686.1 |
|  | FLATFOOT BILATERAL | 734.0 |
|  | GAIT PROBLEM | 781.2 |
|  | GENU VARUS - BOWLEGS CONGENITAL | 736.42 |
|  | HYPERMOBILITY SYNDROME | 728.5 |
|  | INTOEING | 735.8 |
|  | INTOEING BILATERAL | 735.8 |
|  | INTOEING RIGHT | 735.8 |
|  | MACROCRANIA | 756.0 |
|  | METATARSUS ADDUCTUS | 754.53 |
|  | METATARSUS ADDUCTUS CONGENITAL | 754.53 |
|  | OSTEOGENESIS IMPERFECTA | 756.51 |
|  | OUTTOEING | 735.8 |
|  | OUTTOEING LEFT | 735.8 |
|  | PECTUS CARINATUM ACQUIRED | 754.82 |
|  | PECTUS EXCAVATUM | 754.81 |
|  | PECTUS EXCAVATUM CONGENITAL | 754.81 |
|  | PIGEON-TOE | 735.5 |
|  | PLAGIOCEPHALY | 754.0 |
|  | SCOLIOSIS | 737.30 |
|  | SHORT LEG CONGENITAL | 755.30 |
|  | SYNDACTYLY | 755.10 |
|  | SYNDACTYLY TOES WITHOUT BONE FUSION | 755.10 |
|  | TALIPES | 754.51 |
| ORTHOPEDIC-FRACTURE | FRACTURE ANKLE | 824.8 |
|  | FRACTURE CLAVICLE | 810.0 |
|  | FRACTURE CLAVICLE CLOSED | 810.00 |
|  | FRACTURE FACIAL BONES | 802.0 |
|  | FRACTURE FEMUR PERTROCHANTERIC CLOSED | 820.22 |
|  | FRACTURE HUMERUS SUPRACONDYLAR CLOSED | 812.41 |
|  | FRACTURE METACARPALS CLOSED | 815.00 |
|  | FRACTURE PHALANX/PHALANGES CLOSED | 816.00 |
|  | FRACTURE RADIUS | 813.0 |
|  | FRACTURE RADIUS NECK CLOSED | 813.06 |
|  | FRACTURE SKULL | 803.00 |
|  | FRACTURE TIBIA AND FIBULA SHAFT CLOSED | 823.2 |
|  | FRACTURE TIBIA WITH FIBULA CLOSED | 823.22 |
|  | FRACTURE ULNA SHAFT ALONE CLOSED | 813.82 |
| PSYCHIATRIC | ATTENTION DEFICIT DISORDER | 314.00 |
|  | ATTENTION DEFICIT DISORDER OF CHILDHOOD | 314.00 |
|  | ATTENTION DEFICIT DISORDER WITH HYPERACTIVITY | 314.01 |
|  | BEHAVIORAL PROBLEMS | V40.0 |
|  | EMOTIONAL DISTURBANCE CHILDHOOD | 313.9 |
|  | INTELLECTUAL DISABILITIES | 319.0 |
|  | MENTAL AND BEHAVIORAL PROBLEMS | V40.9 |
|  | MENTAL RETARDATION | 319.0 |
|  | RETARDATION MENTAL | 319.0 |
|  | SLOW DEVELOPMENT | 315.9 |
|  | SPEECH DEFECT TRAINING | 784.59 |
|  | SPEECH DISTURBANCE | 315.39 |
|  | SPEECH OR LANGUAGE DISORDERS | 315.39 |
| RENAL | CYST KIDNEY | 753.10 |
|  | FUSION OF KIDNEY | 753.3 |
|  | HYDRONEPHROSIS | 591.0 |
|  | HYDROURETER | 593.5 |
| RESPIRATORY | ASTHMA | 493.9 |
|  | BREATH-HOLDING ATTACK | 786.9 |
|  | BRONCHIAL ASTHMA | 493.0 |
|  | BRONCHOPULMONARY DYSPLASIA | 770.7 |
|  | COUGH | 786.2 |
|  | LARYNGOMALACIA/CONGENITAL | 748.3 |
|  | POLYPS NASAL | 471.0 |
|  | RESPIRATORY DISTRESS SYNDROME | 769.0 |
|  | RESPIRATORY DISTRESS SYNDROME OF NEWBORN | 769.0 |
|  | RHINORRHEA | 349.81 |
|  | SNORING | 786.09 |
|  | STRIDOR | 786.1 |
|  | STRIDOR CONGENITAL | 786.1 |
|  | UPPER AIRWAY OBSTRUCTION | 465.9 |
|  | WHEEZING | 786.07 |
|  | WHEEZING BABY SYNDROME | 786.07 |
| UROLOGICAL | ATROPHY OF TESTIS | 608.3 |
|  | CRYPTORCHISM | 752.51 |
|  | DIVERTICULUM OF BLADDER | 596.3 |
|  | HYDROCELE | 603.9 |
|  | HYPOSPADIA | 752.61 |
|  | HYPOSPADIAS | 752.61 |
|  | MEATAL STENOSIS | 753.6 |
|  | NEPHROLITHIASIS | 274.11 |
|  | PHIMOSIS | 605.0 |
|  | TORSION OF TESTIS | 608.20 |
|  | UNDESC.TESTICLE/CRYPTORCHISM | 752.51 |
|  | UNDESCENDED TESTIS | 752.51 |
|  | UNRETRACTABLE FORESKIN | 605.0 |
|  | URETEROPELVIC JUNCTION STENOSIS UVJ STENOSIS | 753.21 |

**Table S3:** **Diagnoses given by the primary physician in the community clinic, based on pathophysiological classification stratified by sex.**

|  | ASD  N=459 | | w/o ASD  N=2285 | | OR (95% CI) | | Breslow-Day test of OR homogeneity  p-value |
| --- | --- | --- | --- | --- | --- | --- | --- |
|  | Boys  N=371 | Girls  N=88 | Boys  N=1845 | Girls  N=440 | Boys | Girls |  |
| Allergy/ hypersensitivity | 63 (17%) | 9 (10.2%) | 215 (11.7%) | 29 (6.6%) | 1.55 (1.14-2.11) | 1.62 (0.736-3.542) | 0.925 |
| Anemia | 7 (1.9%) | 5 (5.7%) | 47 (2.5%) | 8 (1.8%) | 0.74 (0.33-1.64) | 3.25 (1.04-10.19) | **0.029** |
| Congenital | 46 (12.4%) | 8 (9.1%) | 124 (6.7%) | 18 (4.1%) | 1.96 (1.37-2.81) | 2.34 (0.99-5.58) | 0.711 |
| Epilepsy | 9 (2.4%) | 2 (2.3%) | 24 (1.3%) | 3 (0.7%) | 1.89 (0.87-4.09) | 3.39 (0.56-20.58) | 0.695 |
| Hearing impairment | 11 (3%) | 2 (2.3%) | 12 (0.7%) | 2 (0.5%) | 4.67 (2.05-10.66) | 5.09 (0.71-36.65) | 0.936 |
| Infectious | 16 (4.3%) | 4 (4.5%) | 51 (2.8%) | 5 (1.1%) | 1.59 (0.89-2.81) | 4.143 (1.09-15.75) | 0.184 |
| Overweight | 33 (8.9) | 6 (6.8%) | 10 (6%) | 28 (6.4%) | 1.54 (1.03-2.31) | 1.08 (0.43-2.68) | 0.481 |
| Sleep apnea | 4 (1.1%) | 1 (1.1%) | 15 (0.8%) | 3 (0.7%) | 1.33 (0.44-4.03) | 1.67 (0.17-16.29 | 0.858 |
| Trauma | 5 (1.3%) | 0 (0%) | 15 (0.8%) | 3 (0.7%) | 1.67 (0.60-4.14) | 0.83 (0.80-0.87) | 0.327 |
| Visual impairment | 3 (0.8%) | 2 (2.3%) | 8 (0.4%) | 1 (0.2%) | 1.87 (0.49-7.09) | 10.21 (1.92-113.85) | 0.203 |

The differences in diagnoses between children with and without ASD were evaluated via Pearson's Chi-square or Fisher exact tests. Statistically significant differences (p<0.05) are highlighted in bold font.

**Table S4:** **Diagnoses given by the primary physician in the community clinic, based on pathophysiological classification stratified by ethnicity (Jewish/Bedouin).**

|  | ASD  N=459 | | | w/o ASD  N=2285 | | | OR (95% CI) | | Breslow-Day test of OR homogeneity  p-value |
| --- | --- | --- | --- | --- | --- | --- | --- | --- | --- |
|  | Bedouin  N=133 | Jewish  N=326 | Bedouin  N=133 | | Jewish  N=326 | Bedouin | | Jewish |  |
| Allergy/ hypersensitivity | 20 (15%) | 52 (16%) | 41 (6,2%) | | 203 (12.5%) | 2.69 (1.52-4.77) | | 1.33 (0.95-1.84) | **0.033** |
| Anemia | 8(6%) | 4 (1.2%) | 30 (4.5%) | | 25 (1.5%) | 1.36 (0.61-3.02) | | 0.79 (0.27-2.29) | 0.427 |
| Congenital | 22 (16.5%) | 32 (9.8%) | 42 (6.3%) | | 100 (6.2%) | 2.94 (1.69-5.12) | | 1.654 (1.09-2.51) | 0.103 |
| Epilepsy | 3 (2.3%) | 8 (2.5%) | 6 (0.9%) | | 21 (1.35) | 2.54 (0.63-10.26) | | 1.92 (0.84-4.36) | 0.712 |
| Hearing impairment | 5 (3.8%) | 8 (2.5%) | 4 (0.6%) | | 10 (0.6%) | 6.46 (1.71-24.37) | | 4.05 (1.59-10.34) | 0.573 |
| Infectious | 6 (4.5%) | 14 (4.3%) | 16 (2.4%) | | 40 (2.5%) | 1.92 (0.74-4.92) | | 1.77 (0.95-3.30) | 0.893 |
| Overweight | 13 (9.8%) | 26 (8%) | 31 (4.7%) | | 107 (6.6%) | 2.22 (1.13-4.36) | | 1.23 (0.78-1.91) | 0.149 |
| Sleep apnea | 2 (1.5%) | 3 (0.9%) | 5 (0.8%) | | 13 (0.8%) | 2.02 (0.39-10.50) | | 1.15 (0.33-4.05) | 0.593 |
| Trauma | 1 (0.8%) | 4 (1.2%) | 3 (0.5%) | | 15 (0.9%) | 1.67 (0.17-16.20) | | 1.33 (0.44-4.03) | 0.859 |
| Visual impairment | 1 (0.8%) | 4 (1.2%) | 2 (0.3%) | | 7 (0.4%) | 2.51 (0.23-27.90) | | 2.86 (0.83-9.84) | 0.924 |

The differences in diagnoses between children with and without ASD were evaluated via Pearson's Chi-square or Fisher exact tests. Statistically significant differences (p<0.05) are highlighted in bold font.

**Table S5:** **Diagnoses given by the primary physician in the community clinic, based on anatomical/systemic classification stratified by sex.**

|  | ASD  N=459 | | | w/o ASD  N=2285 | | | OR (95% CI) | | Breslow-Day test of OR homogeneity  p-value |
| --- | --- | --- | --- | --- | --- | --- | --- | --- | --- |
|  | Boys  N=371 | Girls  N=88 | Boys  N=1845 | | Girls  N=440 | Boys | | Girls |  |
| Abdominal wall defect | 3 (0.8%) | 2 (2.3%) | 34 (1.8%) | | 4 (0.9%) | 0.43 (0.13-1.42) | | 2.54 (0.46-14.06) | 0.071 |
| Adenoid / Tonsils | 28 (7.5%) | 1 (1.1%) | 78 (4.2%) | | 13 (3%) | 1.85 (1.18-2.89) | | 0.378 (0.05-2.92) | 0.105 |
| Auricular diseases | 6 (1.6%) | 1 (1.1%) | 7 (0.4%) | | 0 (0%) | 4.32 (1.44-12.92) | | - |  |
| Cardiovascular | 24 (6.5%) | 2 (2.3%) | 62 (3.4%) | | 13 (3%) | 1.99 (1.23-3.23) | | 0.76 (0.17-3.45) | **0.019** |
| Dermatological | 7 (1.9%) | 2 (2.3%) | 13 (0.7%) | | 5 (1.1%) | 2.710 (1.07-6.84) | | 2.02 (0.39-10.60) | 0.762 |
| Endocrinological | 37 (10%) | 9 (10.2%) | 132 (7.25) | | 28 (6.4%) | 1.44 (0.98-2.11) | | 1.68 (0.76-3.69) | 0.731 |
| Gastrointestinal | 19 (5.1%) | 6 (6.8%) | 57 (3.1%) | | 12 (2.7%) | 1.69 (1.00-2.88) | | 2.61 (0.95-7.15) | 0.455 |
| Hematological | 13 (3.5%) | 6 (6.8%) | 67 (3.6%) | | 13 (3%) | 0.96 (0.53-1.76) | | 2.403 (0.89-6.51) | 0.119 |
| Neurological | 60 (16.2%) | 16 (18.2%) | 47 (2.5%) | | 7 (1.6%) | 7.38 (4.95-11.01) | | 13.75 (5.46-34.58) | 0.222 |
| Neuro-ophthalmological | 5 (1.3%) | 2 (2.35) | 7 (0.4%) | | 1 (0.2%) | 3.59 (1.13-11.36) | | 10.21 (0.92-113.85) | 0.432 |
| Neuromuscular | 3 (0.8%) | 1 (1.1%) | 8 (0.4%) | | 1 (0.2%) | 1.87 (0.49-7.09) | | 5.05 (0.31-81.45) | 0.519 |
| Ophthalmological | 9 (2.4%) | 3 (3.4%) | 15 (0.8%) | | 3 (0.7%) | 3.03 (1.32-6.98) | | 5.14 (1.02-25.90) | 0.568 |
| Orthopedic | 11 (3%) | 1 (1.1%) | 23 (1.2%) | | 5 (1.1%) | 2.42 (1.17-5.01) | | 1.00 (0.12-8.675) | 0.436 |
| Orthopedic-fracture | 4 (1.1%) | 0 | 9 (0.5%) | | 2 (0.5%) | 2.22 (0.68-7.26) | | 0.83 (0.80-0.87) | 0.359 |
| Psychiatric | 46 (12.4%) | 7 (8%) | 46 (2.5%) | | 7 (1.6%) | 5.54 (3.62-8.47) | | 5.35 (1.83-15.65) | 0.953 |
| Renal | 1 (0.3%) | 0 | 13 (0.7%) | | 1 (0.2%) | 0.38 (0.05-2.92) | | 0.83 (0.80-0.87) | 0.783 |
| Respiratory | 32 (8.6%) | 6 (6.8%) | 125 (6.8%) | | 13 (3%) | 1.30(0.87-1.95) | | 2.40 (0.898-6.51) | 0.257 |
| Urological | 16 (4.3%) | 0 | 60 (3.3%) | | 0 | 1.34 (0.76-2.36) | | - | - |

The differences in diagnoses between children with and without ASD were evaluated via Pearson's Chi-square or Fisher exact tests. Statistically significant differences (p<0.05) are highlighted in bold font.

|  | ASD  N=459 | | w/o ASD  N=2285 | | OR (95% CI) | | Breslow-Day test of OR homogeneity  p-value |
| --- | --- | --- | --- | --- | --- | --- | --- |
|  | Bedouin  N=133 | Jewish  N=326 | Bedouin  N=133 | Jewish  N=326 | Bedouin | Jewish |  |
| Abdominal wall defect | 1 (0.8%) | 4 (1.2%) | 15 (2.3%) | 23 (1.4%) | 0.33 (0.04-2.51) | 0.86 (0.30-2.51) | 0.396 |
| Adenoid / Tonsils | 11 (8.3%) | 18 (5.5%) | 23 (3.5%) | 68 (4.2%) | 2.52 (1.20-5.30) | 1.33 (0.78-2.28) | 0.171 |
| Auricular diseases | 3 (2.3%) | 4 (1.2%) | 1 (0.2%) | 6 (0.4%) | 15.32 (1.58-148.46) | 3.34 (0.94-11.91) | 0.233 |
| Cardiovascular | 11 (8.3%) | 15 (4.6%) | 16 (2.4%) | 59 (3.6%) | 3.66 (1.66-8.07) | 1.28 (0.72-2.28) | **0.032** |
| Dermatological | 1 (0.8%) | 8 (2.5%) | 4 (0.6%) | 14 (0.9%) | 1.25 (0.14-11.29) | 2.89 (1.20-6.94) | 0.481 |
| Endocrinological | 14 (10.5%) | 32 (9.8%) | 37 (5.6%) | 123 (7.6%) | 2.00 (1.05-3.81) | 1.33 (0.88-1.99) | 0.291 |
| Gastrointestinal | 10 (7.5%) | 15 (4.6%) | 12 (1.8%) | 57 (3.5%) | 4.42 (1.87-10.50) | 1.32 (0.74-2.37) | **0.019** |
| Hematological | 11 (8.3%) | 8 (2.5%) | 41 (6.2%) | 39 (2.4%) | 1.37 (0.69-2.75) | 1.02 (0.47-2.20) | 0.574 |
| Neurological | 36 (27.1%) | 40 (12.3%) | 15 (2.3%) | 39 (2.4%) | 16.08 (8.49-30.47) | 5.67 (3.58-8.97) | **0.009** |
| Neuro-ophthalmological | 1 (0.8%) | 6 (1.8%) | 2 (0.3%) | 6 (0.4%) | 2.51 (0.23-27.90) | 5.04 (1.62-15.74) | 0.604 |
| Neuromuscular | 1 (0.8%) | 3 (0.9%) | 2 (0.3%) | 7 (0.4%) | 2.51 (0.23-27.90) | 2.14 (0.55-8.32) | 0.910 |
| Ophthalmological | 5 (3.8%) | 7 (2.1%) | 3 (0.5%) | 15 (0.9%) | 8.62 (2.03-36.52) | 2.35 (0.95-5.81) | 0.125 |
| Orthopedic | 4 (3%) | 8 (2.5%) | 9 (1.4%) | 19 (1.2%) | 2.26 (0.69-7.45) | 2.12 (0.92-4.895) | 0.931 |
| Orthopedic-fracture | 1 (0.8%) | 3 (0.9%) | 1 (0.2%) | 10 (0.6%) | 5.03 (0.31-80.93) | 1.50 (0.41-5.46) | 0.420 |
| Psychiatric | 27 (20.3%) | 26 (8.0%) | 9 (1.4%) | 44 (2.7%) | 18.57 (8.50-40.57) | 3.10 (1.88-5.12) | **<0.001** |
| Renal | 1 (0.8%) | 0 | 6 (0.9%) | 8 (0.5%) | 0.83 (0.10-6.97) | - | - |
| Respiratory | 10 (7.5%) | 28 (8.6%) | 23 (3.5%) | 115 (7.1%) | 2.27 (1.05-4.89) | 1.23 (0.90-1.90) | 0.169 |
| Urological | 8 (6%) | 8 (2.5%) | 16 (2.4%) | 44 (2.7%) | 2.60 (1.09-6.20) | 0.90 (0.42-1.93) | 0.067 |

**Table S6:** **Diagnoses given by the primary physician in the community clinic, based on anatomical/systemic classification stratified by ethnicity (Jewish/Bedouin).**

The differences in diagnoses between children with and without ASD were evaluated via Pearson's Chi-square or Fisher exact tests. Statistically significant differences (p<0.05) are highlighted in bold font.
